# Innovation through internationalization: A systematic review and research agenda

**DOI:** 10.1007/s10490-022-09814-z

**Published:** 2022-03-28

**Authors:** Jian Du, Shan Zhu, Wen Helena Li

**Affiliations:** 1 School of Management, School of Management Building, Zhejiang University, Room 818, Hangzhou, 310058 Zhejiang China; 2 School of Management, Zhejiang University, Room 811-14, School of Management Building, Hangzhou, 310058 Zhejiang China; 3 UTS Business School, University of Technology Sydney, Sydney, NSW Australia

**Keywords:** Internationalization, Innovation, Systematic literature review

## Abstract

In this paper we perform a systematic literature review of the diverse and somewhat fragmented current state of research on firms’ internationalization and innovation. We analyze 207 key works from 1989 through 2020 and synthesize them into an internationalization process framework that conceptually maps key internationalization-related antecedents and moderators that influence innovation behaviors and outcomes. Through an internationalization process framework, we categorize existing relevant studies into three key stages: (a) the pre-internationalization stage, (b) the internationalization entry stage, and (c) the post-internationalization stage. Furthermore, we review how firms’ various strategic decisions and operations in different stages influence their innovations by elaborating the moderating role of external country/region institutions and firm internal characteristics. Building on this review, we provide suggestions for future research to advance the developments of this domain.

Innovation is of great importance for firms’ long-term sustainability and global competitiveness (Kafouros et al., 2008). It is impossible to innovate in a sustainable manner within a firm’s home country only, given that each country has its own limitations and constraints (Anand et al., 2021). Since the early 1980s, leveraging multiple innovation resources inside and outside of firms’ home countries has become an essential source for firms to develop innovation capabilities, and a growing number of firms have documented the role of internationalization in their innovation performance (Papanastassiou et al., 2020; Zhao et al., 2021). Also, to better understand the strategic decisions and outcomes behind whether and how firms augment their innovation capabilities through the internationalization process, scholars have produced a vast amount of work (e.g., Ananthram & Chan, 2021; Hitt et al., 1997; Kafouros et al., 2008; Phene & Almeida, 2008; Xie & Li, 2018; Zahra et al., 2000).

In this paper, we aim to review the literature on the role of internationalization in firms’ innovation, covering publications from 1989 to 2020, for several purposes. First, despite a sustained and growing interest by the international business (IB) community in linking firms’ internationalization with innovation, we find this domain highly fragmented, with scattered findings and diversified theoretical perspectives; a systematic review is absent thus far. Only recently, Christofi et al. (2019) and Papanastassiou et al. (2020) have attempted to address this gap. However, the former focused exclusively on the impact of micro level factors on technological innovation in the context of cross-border acquisitions, whereas the latter shed considerable light on multinational enterprises’ (MNEs) global research and development (R&D) over the past 50 years through historically changing perspectives. Thus, a more systematic review covering firms’ various internationalization strategies and diverse innovation activities is needed.

Second, inconsistent findings persist regarding the relationship between internationalization and innovation. For instance, there are mixed results on whether firms’ diversity of international locations leads to positive innovation outcomes such as new products (Wu & Park, 2017; Zahra et al., 2000) and whether cross-border acquisition triggers subsidiaries’ innovation behavior such as R&D investments (Bertrand, 2009; Hitt et al., 1991). Moreover, scholars also debate whether subsidiaries’ autonomy can improve innovation outcomes within MNEs (Beugelsdijk & Jindra, 2018; Mudambi et al., 2007). These disagreements call for synthesizing and analyzing the literature to identify consensus and controversies.

Third, global contexts are now undergoing unprecedented political, economic, and social turbulence from various sources, including the “global war” on intellectual property, the rapid development of the digital economy, the COVID-19 pandemic, and trade tensions and protectionism. These pose significant challenges to firms’ processes of building innovation capability (Bahl et al., 2021; Petricevic & Teece, 2019; Sun et al., 2021; Yan et al., 2021), which may call into question prior research findings and serve as an opportunity to revise our existing theories and findings. Without a systematic review of our current knowledge, it is hard for scholars to determine what needs to be revised in rapidly changing environments.

To address the aforementioned gaps, we have integrated current knowledge by surveying the literature on the impact of firms’ internationalization on their innovation. Using systematic literature review (SLR) methodology, we reviewed and coded 207 papers published in a wide range of IB, innovation, and management journals from 1989 to 2020. Through an internationalization process framework, we categorized existing relevant studies into three key stages: (a) the pre-internationalization stage, (b) the internationalization entry stage, and (c) the post-internationalization stage. We further conducted a systematic analysis on whether and how firms’ internationalization influences their innovation behaviors (e.g., R&D investment, R&D alliance formation, and R&D site selection) and innovation outcomes (e.g., patent, product and process). Then we elaborated the contingent role of external country/region institutions and firms’ internal characteristics in this relationship. Furthermore, we identified several research gaps and provided a set of suggestions for future research to advance the developments in this domain.

## Methodology

**Fig. 1:**
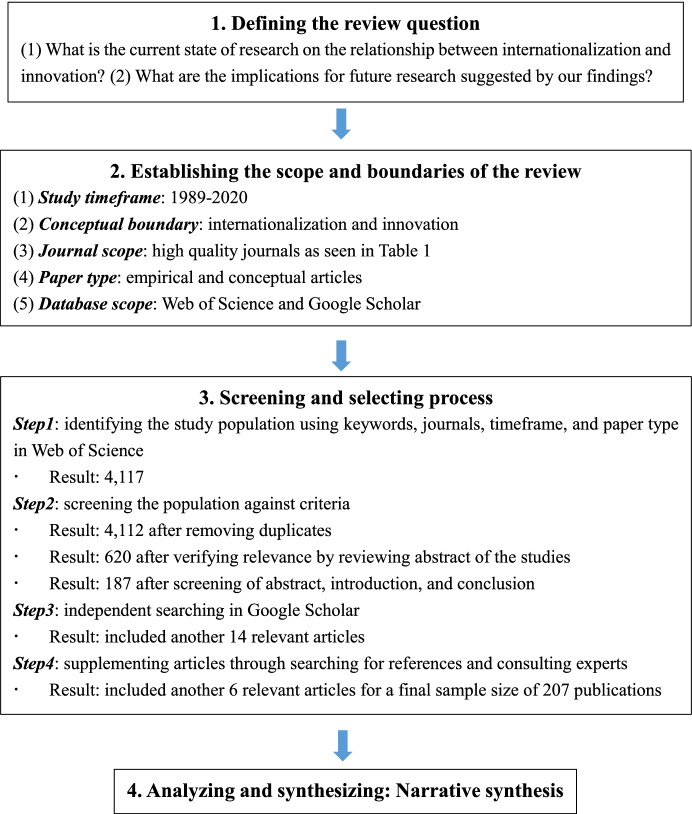
Summary of the systematic review methodology

In this study, we followed the multistep review approach of Denyer and Tranfield (2009) to conduct our SLR of published works on whether and how firms’ internationalization influences their innovation. This methodology provides a rigorous and replicable way to identify, screen, select, and analyze existing literature, and it ensures robustness by eliminating subjectivity in data collection and analysis. Specifically, this approach involves four steps: (a) defining the review questions, (b) establishing the scope and boundary of the review, (c) screening and selecting process, and (d) analyzing and synthesizing. Figure 1 summarizes our four-step iterative process.

### Defining the review question

The ways firms’ internationalization affects their innovation have drawn much attention and produced extensive studies. In our paper, we have tried to build a comprehensive framework that can provide insights and extend our understanding of the essence of this domain and its underlying mechanisms. Thus, our SLR is driven by the following two research questions: *What is the current state of research on the relationship between internationalization and innovation? What implications for future research do our findings suggest?*

### Establishing the scope and boundary of the review

We have determined the exclusion and inclusion criteria for our review to establish its scope and boundaries. First, we set our time frame from 1989 to 2020. We chose 1989 as our starting year because, since the 1980s, leveraging knowledge and resources outside of firms’ home countries has been thought to an important source for developing innovation capabilities (Papanastassiou et al., 2020).

Second, we defined the conceptual boundaries for two key terms: internationalization and innovation. “Internationalization” is a process by which firms gradually increase their international involvement and is the product of a series of incremental decisions (Johanson & Vahlne, 1977). Firms’ internationalization process has been conceptualized as a set of key decisions, including location, entry mode, investment amount, and control and management of the foreign operation (Beugelsdijk et al., 2017). As defined in the European Commission’s *Green Paper of Innovation* (Europeu, 1995), “innovation” is production or adoption, assimilation, and exploitation of a value-added novelty in economic and social spheres; renewal and enlargement of products, services, and markets; development of new methods of production; and establishment of new management systems (pp. 1–2). For our review, we adopted the commonly used classifications for firm-level innovations: innovation behavior and innovation outcome (Adams et al., 2006). Innovation behavior is defined and measured as a firm’s strategic activities and decisions dedicated to the exploration and exploitation of new opportunities, such as R&D investment, R&D site selection, and R&D alliance formation (Duran et al., 2016). Newly or significantly improved patents, products, and processes are the most studied forms of innovation outcomes (Hagedoorn & Cloodt, 2003). Our paper highlights the differences between these two dependent variables used in the literature to more comprehensively summarize this domain. Under the conceptual boundaries, we initially clarified two keywords: “Internation*” and “Innovat*.” Then, we referred to the frequently cited reviews in the fields of internationalization and innovation and consulted relevant scholars to supplement the pool of keywords (Beugelsdijk et al., 2017; Duran et al., 2016; Hagedoorn & Cloodt, 2003). In terms of internationalization, we added keywords such as “global,” “foreign,” and “cross-border,” and for innovation, we added “R&D,” “technology,” “knowledge,” and “learning”.^1^

Third, we considered peer-reviewed articles only, excluding book reviews, magazines, editorials, and interviews to ensure the highest quality and scholarly standards (Newbert, 2007). To ensure our comprehensive coverage of journals, we modelled the journal selection process on two related review articles (Christofi et al., 2019; Papanastassiou et al., 2020) and also included other journals ranked number three and higher in the list of the Academic Journal Quality Guide developed by the Association of Business Schools (ABS). We identified 36 journals (as seen in Table 1). Finally, we used Web of Science and Google Scholar databases to search for articles. We chose Web of Science as our primary search source for this step because it provides excellent journal coverage of the relevant disciplines. Moreover, given that Google Scholar follows a different algorithm that generally has broader coverage than Web of Science, we also conducted an independent search in Google Scholar to confirm our original search results. 2

**Table 1:** Top business journals by focused area searched for the literature review

1 IB journals	3 Management Journals	4 Innovation journals	6 Marketing journals
Asia Pacific Journal of Management	Academy of Management Journal	Journal of Product Innovation Management	European Journal of Marketing
International Business Review	Academy of Management Review	R and D Management	Journal of International Marketing
Journal of International Business Studies	Administrative Science Quarterly	Research Policy	Journal of Marketing
Journal of International Management	British Journal of Management	Technovation	7 Social journals
Journal of World Business	European Management Review	5 Entrepreneurship journals	Industrial and Corporate Change
Management International Review	Journal of Business Research	Entrepreneurship, Theory and Practice	Technological Forecasting & Social Change
2 Strategy journals	Journal of Management	International Small Business Journal	World Development
Global Strategy Journal	Journal of Management Studies	Journal of Business Venturing	8 Regional journal
Strategic Management Journal	Long Range Planning	Strategic Entrepreneurship Journal	Regional Studies
Strategic Organization	Management and Organization Review		
	Management Science		
	Organization Science		

### Screening and selecting process

This step was intended to identify, screen, and select suitable studies to help answer our review questions. First, we conducted the initial search in the Web of Science database with the keywords and journals to identify articles published between 1989 and 2020, and we identified 4,117 articles as potentially relevant for analysis. Second, we screened the 4,117 articles against the defined criteria. We reduced the sample to 4112 by eliminating duplicates, and then scrutinized the 4112 articles based on the fit-for-purpose criteria (Rashman et al., 2009) by reviewing the abstracts of the studies. Given that the objective of this review was to synthesize the literature on the ways firms’ internationalization affect their innovation, we designed the fit-for-purpose criteria to include studies of whether the internationalization of firms was directly and explicitly linked to their innovation behaviors or outcomes and what kind of context applied to the relationship. This step yielded 620 studies. Next, we examined the introductions and conclusions of those studies to further reduce the 620 papers to 187 highly relevant papers. By means of an additional independent search in Google Scholar, we added another 14 relevant articles. Last, we conducted an additional step to ensure that we did not omit any relevant articles. We not only checked the references for the studies that we identified through the above steps, but also consulted experts again to search for additional relevant papers. This step yielded six articles. The final sample size of 207 articles was viable for a systematic review.

### Analyzing and synthesizing

We first used the bibliometric method to make a descriptive analysis of the identified articles and then conducted a narrative synthesis to investigate and combine the 207 studies to identify prevalent research themes and subthemes. A narrative synthesis uses words and texts to summarize and interpret results, aiming to review and synthesize multiple studies systematically (Popay et al., 2006). Different from methodically emphasizing the effectiveness of a particular intervention (e.g., meta-analysis), narrative synthesis focuses on a wide range of issues and aims to synthesize one domain. As an established research domain, whether and how firms’ internationalization influences their innovations will include multiple streams and a broad set of fragmented problems, in which a narrative synthesis can serve as a more effective method.

In the beginning, we coded the stage of the internationalization process, the type of innovation behaviors and outcomes, the theoretical perspective, the study’s research context, and the methodology of each study. We then designed a worksheet to record this information and scrutinized it for potential errors. After that, we adopted the bibliometric method to conduct statistical and descriptive analyses of patterns that appeared in publications in the following section. Next, we arranged these studies into themes by deploying line-by-line coding. Eventually, we provided a comprehensive framework that would fit our review questions and logically integrate the disparate results.

## Bibliometric findings

**Fig. 2:**
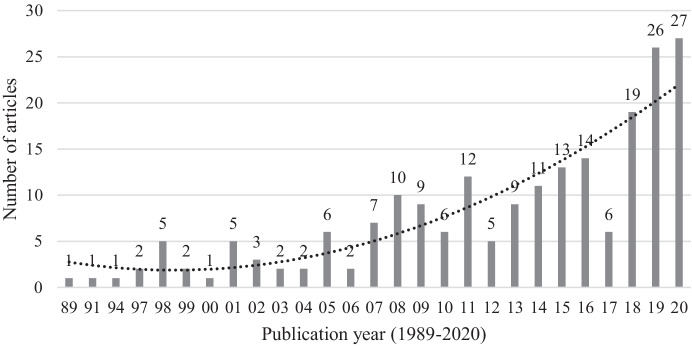
Number of articles year on year (1989–2020)

**Fig. 3:**
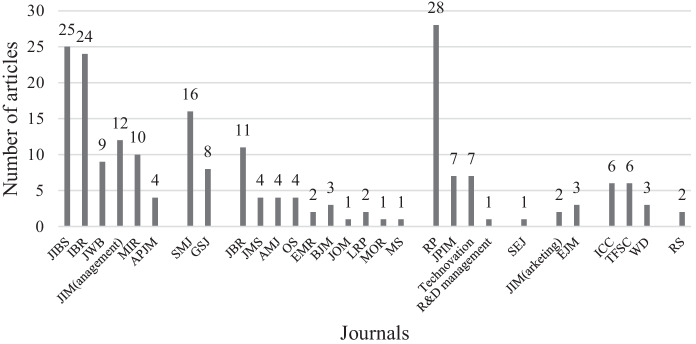
Distribution of articles across journals (1989–2020)

In Fig. 2, we present the number of articles published each year on internationalization and innovation, which clearly shows a steadily increasing trend since one article in 1989, reaching 27 articles in 2020. In Fig. 3, we show the distribution of journals from 1989 to 2020. As expected, the innovation journal *Research Policy* and the IB journal *Journal of International Business Studies* have the highest number of articles among the top journals we surveyed, with 28 and 25 articles, respectively.

In Table 2, we list the theories used in our identified articles. Most scholars (*n* = 129, 62.32%) used a single theory or perspective, in which organizational learning theory, institutional theory, network theory (including embeddedness theory), and knowledge-based view predominated. Eighteen studies involved multiple theoretical perspectives, and most authors tended to integrate one perspective with a resource-based view (including dynamic capabilities), knowledge-based view, or institutional theory. Table 3 is a summary of the top five theories in the domain.

**Table 2:** Theoretical approaches in internationalization-innovation research

Theory/perspective	Total number
Single theory/ perspective	
Organizational learning theory	26
Institution theory/institution-based view	19
Network theory (including embeddedness perspective)	16
Knowledge-based view (KBV)	13
Resource-based view (RBV) (including dynamic capability)	9
Ownership, location & internalization (OLI)	7
Social capital theory	6
Agency theory	4
Contingency theory	4
Transaction cost economics (TCE)	3
Upper echelon theory	3
Uppsala model	2
Organization ecology theory	2
Information processing theory	2
Other theories	13
Total	129
Multiple theories/ perspectives	
TCE & KBV/RBV/institutional theory/network theory	4
Network theory/embeddedness theory & KBV/RBV/institutional theory	5
Organizational learning theory & KBV/RBV	3
RBV/KBV/institutional theory	1
Other theories	5
Total	18
No specified theory	60
Total	207

**Table 3:** Mapping the landscape of the top five theories in the internationalization and innovation domain

Theory	Frequency	How theory is used	Examples
Organizational learning theory	26	1. Organizations learn in different ways depending on their learning ability, prior experiences, and the knowledge base they have developed. 2. MNEs can learn and develop innovations from deep interactions with foreign stakeholders. 3. MNEs’ innovation is linked to their ability to recognize the value of new information, assimilate it, and apply it to commercial ends.	Zahra et al. (2000); Xie and Li (2015); Piperopoulos et al. (2018)
Institution theory/ institution-based view	19	1. The institutional development in one country and the institutional difference between home and host countries both can affect MNEs’ innovation by influencing the effectiveness of their learning. 2. MNEs locate their R&D activities in an institutional context defined by certain rules, norms, and values.	Wu et al. (2015); Xie and Li (2018)
Network theory (including embeddedness perspective)	16	1. Interpersonal and interorganizational networks provide channels for knowledge flows. 2. MNEs’ relational embeddedness encourages more information and knowledge exchange and has lock-in effects as well.	Williams and Du (2014); Isaac et al. (2019)
Knowledge-based view	13	Linking knowledge characteristics with MNEs’ innovation, MNEs can transfer and acquire diversified knowledge from different countries and kinds of collaborators for their innovation.	Almeida and Phene (2004); Hsieh et al. (2018)
Resource-based view (including dynamic capability)	9	1. Strategic resources serving as MNEs’ competitive advantages are valuable, rare, and inimitable. MNEs can integrate and acquire them from global markets and achieve innovations. 2. MNEs’ ability to innovate is closely linked to their ability to adapt, integrate, and reconfigure knowledge globally.	Kotabe et al. (2007); Michailova and Zhan (2015)

In Table 4, we provide a summary of the research methods used in our reviewed articles. The main method employed was quantitative research (*n* = 181, 87.44%), in which 71.82% of the articles involved secondary data, and 28.18% involved survey data. Descriptive/theoretical analysis (*n* = 15, 7.25%) and case studies (*n* = 11, 5.31%) were less common. In Table 5, we show the distribution of countries and regions.

**Table 4:** Research method

Methodology	Total number	Percentage (%)
Descriptive/theoretical analysis	15	7.25
Case study	11	5.31
Quantitative research		
Survey data	51	24.64
Secondary data	130	62.80
Total	207	100.00

**Table 5:** Countries and regions studies

Country	Total number
Single country	
Developed country	
US	24
Japan	12
Spain	11
German	7
UK	6
Sweden	5
Italy	4
others	5
Developing country	
China	39
India	8
others	5
Multiple economies	
Multiple developed economies	15
Multiple developing economies	2
Not specified and not mentioned	64
Total	207

## Thematic analysis of firms’ internationalization and innovation

**Fig. 4:**
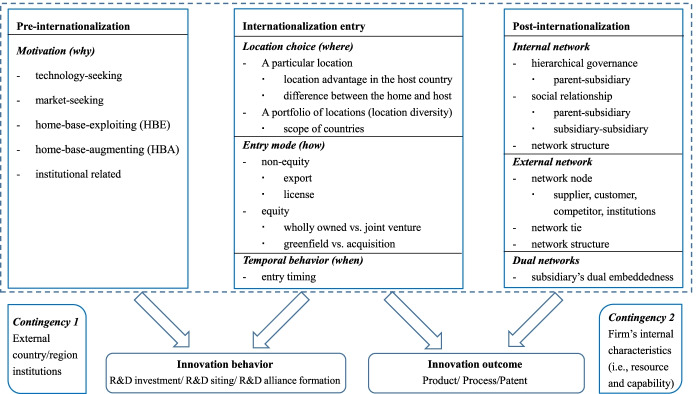
A comprehensive framework between internationalization and innovation

Building on 207 publications from 1989 to 2020 in this domain, we suggest the integrative framework shown in Fig. 4. We followed the concept boundary for internationalization and divided the internationalization process into three stages: the pre-internationalization stage, the internationalization entry stage, and the post-internationalization stage. Various strategic decisions and operations in different stages play important roles in firms’ innovation. Under each stage, whenever applicable, we will review the direct impact of internationalization on innovation behaviors and innovation outcomes separately. Furthermore, the country/region’s institutional environment, where firms are embedded, and firms’ internal characteristics (e.g., capabilities and resources) serve as contingencies for determining the extent to which firms can make use of the advantages of internationalization to achieve innovation.

### Pre-internationalization

The motivations for foreign expansion play direct and important roles in driving firms’ innovation behaviors, especially for R&D site selections. Technology-seeking MNEs tend to locate their research activities in technology-advanced countries to gain access to local research institutions and technological talent and to capture industry knowledge spillover from local competitors (Cantwell & Piscitello, 2005). For market-seeking MNEs whose business mainly depends on customer demand, it is more favorable to arrange product development and design activities in highly dispersed locations and in host countries with a large customer base (Shimizutani & Todo, 2008; von Zedtwitz & Gassmann, 2002). Other scholars compare home-base-exploiting (HBE) and home-base-augmenting (HBA) motivations. HBE MNEs that seek to exploit firm-specific capabilities usually establish overseas R&D activities close to manufacturing facilities and marketing facilities, where they can exploit their own knowledge base to produce new products to meet local demand; HBA MNEs that seek to augment their knowledge base prefer to establish R&D activities close to universities and government laboratories because they can access unique resources and use externalities created by local institutions and firms (Bas & Sierra, 2002; Kuemmerle, 1999). Moreover, scholars have discussed how institutional-related motivations (e.g., escaping from the weak institutional environments in home countries) drive firms to search for overseas R&D sites that can provide access to sophisticated technologies or stronger protections for intellectual property (Cuervo-Cazurra & Ramamurti, 2017). In addition, it is believed that developed multinational enterprises (DMNEs) tend to expand across country borders to exploit their own technological assets and innovative capabilities, whereas emerging multinational enterprises (EMNEs) are deemed to seek overseas strategic assets with the intention of benefiting from proximity to well-developed institutions and key external actors to take advantage of spillovers and develop competitive advantages (Lundan & Dunning, 2008; Luo & Tung, 2007).

### Internationalization entry

At this stage, firms’ strategies, including (a) location choice (i.e., where to enter), (b) entry mode (i.e., how to enter), and (c) temporal behavior (i.e., when to enter), have been found to have a great influence on firms’ innovation behaviors and outcomes.

#### Location choice

The choice of location consists of topics either focusing on a particular location or a portfolio of locations and the dominant theories in explaining its roles in innovation are organizational learning theory, institutional theory, and resource-based views (Hitt et al., 1997; Xie & Li, 2015; Zahra et al., 2000).

The determination of a particular location implies firms’ choice regarding the location features, in which the advantages (e.g., infrastructures, policies, markets, and talents) in a certain host country not only stimulate firms’ innovation behaviors but also support them in producing more innovation outcomes. For example, locating in countries with high levels of protection of intellectual property rights could foster MNEs’ R&D investments because the risk of being imitated is low, and firms can gain more profits from their R&D activities (Ito & Wakasugi, 2007). Locating in developed countries serves as an effective channel for EMNEs to overcome internal resource constraints and to escape from weak institutional environments in the home country, thereby achieving better innovation outcomes such as new products and patents (Fu et al., 2018; Piperopoulos et al., 2018). On the other hand, knowledge originated in and developed for an emerging economy can also become an important source of innovation outcomes for firms in an advanced country (known as reverse innovation). This knowledge can help them cater to major emerging markets and low-priced segments in developed countries (Huang & Li, 2019). Besides location-specific advantages, the difference or distance between a certain host country and the home country is the other location feature, which has influences on firms’ innovation. For instance, Joshi and Lahiri (2015) found an inverted U-shaped relationship between language friction and R&D alliance formation in host countries, because while institutional difference offers diverse knowledge, it incurs higher transaction costs and hinders interactive learning. McCarthy and Aalbers (2016) highlighted the hurdles in distant countries (e.g., transaction costs, agency costs, and communication and knowledge transfer difficulties) and found that geographical distance has negative impacts on innovation outcomes (i.e., new patents).

Another topic is focused on the portfolio of locations, especially the diversity of country portfolios (i.e., location diversity). Most studies under this topic link location diversity with innovation outcomes, with two contrasting arguments. On the one hand, scholars found that location diversity could improve firms’ learning and innovation (Elia et al., 2020; Wu et al., 2015; Zahra et al., 2000). First, it was beneficial for firms to acquire innovative resources (i.e., advanced technology and talent), identify innovative opportunities, and learn from diverse foreign stakeholders across different countries, and such increased knowledge and resource bases could fuel firms’ innovation (Hitt et al., 1997). Second, expansion into diversified international markets provided potential for greater returns on innovation, stimulating firms to innovate further (Hitt et al., 1994). Third, compared to domestic firms, internationally diversified firms faced fierce competition in foreign markets, encouraging them to innovate quickly (Wu et al., 2015). On the other hand, other studies have observed that regardless of the benefits of location diversity for firms’ innovation outcomes, challenges for innovation could increase. From the perspective of institutional theory, firms located in a wider range of countries face greater risks and uncertainties in diverse institutional settings, and firms have to invest more efforts in operational activities to cope with these uncertainties, so minimal attention could be left for innovation activities (Hsu et al., 2015; Wu & Park, 2017). High cooperation and communication costs associated with increased location diversity also make it hard for firms to support their innovation activities effectively (Lahiri, 2010). Moreover, internationally diversified firms faced learning challenges by which they suffered from information overload and had difficulty identifying useful information as increasing complexities arise from having diverse locations (Wu & Park, 2017), which is especially true for EMNEs due to their limited absorptive capacity (Li et al., 2010). Thus, researchers found an inverted U-shaped relationship between location diversity and innovation outcomes (Lahiri, 2010; Wu & Park, 2017).

To address these mixed findings in the relationship between location diversity and innovation, researchers have further explored several firm characteristics. It is agreed that firms’ capabilities, such as dynamic capability and absorptive capacity, can help them generate more innovation outcomes from location diversity through identifying and acquiring externally generated knowledge more effectively (Wu et al., 2016; Xie & Li, 2015). Hsu et al. (2015) found that compared to MNEs without international experience, internationally experienced MNEs gained more benefits and less harm from location diversity because international experience mitigated complexities and uncertainties in the host country. Tsao and Lien (2013) suggested that family MNEs gain more new patents through location diversity than nonfamily MNEs.

#### Entry mode

When entering global markets, another critical step is how to enter, namely, the entry mode decision. Entry mode can be divided into two categories: equity and non-equity, in which the former requires high levels of commitment and control from parent firms (e.g., joint venture, wholly owned ventures, greenfield and acquisition), while the latter needs a low level of commitment and control (e.g., license and export) (Pan & Tse, 2000). Organizational learning theory has been cited most frequently in this topic (Xie & Li, 2015; Zahra et al., 2000).

In terms of innovation behavior, scholars have discussed how entry mode decision affects firms’ R&D investments. As for non-equity mode, Chittoor et al. (2015) believed export could stimulate firms’ R&D investments because firms need to enhance their capabilities through internal R&D investments to grasp a multitude of learning opportunities in export. But the influence of equity mode on innovation behavior is not conclusive. Hitt et al. (1991) found a reduction in R&D intensity for post-acquisition affiliates because acquisitions that needed high degrees of commitment and control appeared to divert financial resources and managers’ attention from R&D investments, whereas Bertrand (2009) proposed that cross-border acquisition could encourage post-acquisition affiliates’ R&D investments because it provided efficiency gains such as generated scale and scope economies to spread the fixed costs over more R&D activities.

For the innovation outcome, most scholars have addressed the fact that both equity and non-equity modes can help firms improve it by accessing diverse foreign knowledge and by learning advanced technologies, while the underlying mechanisms are somewhat different (Cassiman & Golovko, 2011; Piperopoulos et al., 2018; Zahra et al., 2000). For the non-equity mode, such as in export, firms face lower barriers to learn from internationalization because this entry mode requires less sophisticated management skills and involves fewer commitments or risks (Cassiman & Golovko, 2011). Xie and Li (2018) found that export created a channel for domestic firms to learn from foreign partners, and thus export intensity positively affected new product outcomes for Chinese firms. Compared to the non-equity mode, the equity mode provides firms with more complex knowledge sources and learning opportunities through deeper interactions with foreign stakeholders (Guo & Clougherty, 2020; Zahra et al., 2000, 2009). Zahra et al. (2009) found that the greater the involvement of modes of entering foreign markets, the greater the number of new products because firms can increase their exposure to different information sources in the host country.

Further, scholars have compared different equity modes in influencing innovation outcomes. For example, international joint ventures (IJVs) with frequent interactions with JV partners have more diversified knowledge than wholly owned ventures, and Yao et al. (2013) found that knowledge complementarity between JV parties can improve partners’ absorptive capacity, further increasing international JVs’ new product performance. De Noni and Apa (2015) found that greenfield could foster exploitative learning, while cross-border acquisition could lead to exploratory learning that enables the development of new skills and capabilities currently not in the MNEs’ repertoire.

#### Temporal behavior

Firms’ foreign expansion is a dynamic process, in which time matters (Johanson & Vahlne, 1977; Vermeulen & Barkema, 2002). Entry decisions entail the timing and speed of entering particular foreign markets, and scholars have started to discuss how such temporal behaviors influence firm innovations. The timing of entry targets the time lag between the founding of a firm and its initial international expansion, in which international new ventures (INVs) have attracted much attention because they internationalize early in their life cycle, and scholars have found that INVs can leverage learning advantages (i.e., learn quickly and flexibility) to facilitate their knowledge absorption and creation (Zahra et al., 2000). As for the entry speed, defined as the number of foreign expansions undertaken by a firm over a specific period of time (Yang et al., 2017), empirical evidence linking it with innovation is still lacking. Despite scholars’ having found that “sustaining” entry could increase R&D investments and new product outcomes through maintaining their exposure to the global markets to learn more effectively (Huang, 2013), it is still unclear how the speed in sustaining entry influences firms’ innovations.

### Post-internationalization

**Fig. 5:**
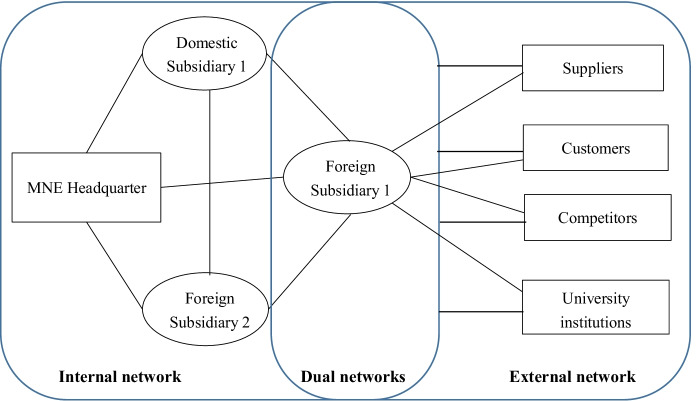
MNEs’ operation in internal, external and dual networks

After expanding into other countries, MNEs should manage their foreign operations through interacting with multiple actors in various complex networks (Johanson & Vahlne, 2009). Networks provide channels for knowledge flows (Bergek & Bruzelius, 2010), and there has been a significant increase in discussing MNEs’ innovation through network perspectives and knowledge-based views (Andersson et al., 2002; Williams & Du, 2014). Thus, we will use a network lens to guide the summary of literature in this section (see Fig. 5). Since a firm’s foreign practices directly influence the final performance that is crucial for a firm’s survival and development (Beugelsdijk et al., 2017; Lundan & Dunning, 2008), scholars concentrated more on the impacts of firms’ foreign operations on the innovation outcomes under this subtheme.

#### MNEs’ internal network

MNEs are composed of a complex network that includes a large number of interconnected internal units, and each unit is highly involved in MNEs’ absorption, use, and generation of knowledge (Bergek & Bruzelius, 2010). The research on MNES’ internal networks mostly addresses how to design proper hierarchical governance between parent and foreign subsidiaries and how to develop parent–subsidiary and subsidiary–subsidiary relationships, which can facilitate knowledge transfer and innovation outcomes.

MNEs establish hierarchical governance to control and coordinate overseas subsidiaries to achieve their global organization objectives, for which one core research question is how the degrees of control from parent firms affect subsidiaries’ innovation outcomes but studies remain controversial. Mudambi et al. (2007) found that the lesser the degree of parent controls, the higher the degree of knowledge creation in subsidiaries because minimal controls positively enhance their own intrinsic inspiration to innovate. However, Beugelsdijk and Jindra (2018) argued that despite higher degrees of new product novelty benefiting from subsidiaries’ autonomy, managerial involvement from headquarters was required as well, because extremely low degrees of control are dangerous that may result in rent-seeking behavior by subsidiary managers, a lack of information exchange and shared identity, and the liability of internal isolation. Moreover, scholars have discussed the types of control modes and found that social control and result control can build a relaxing and educational organizational atmosphere, thereby promoting knowledge flow, stimulating members’ creativity, and finally enhancing subsidiaries’ innovation activities, whereas formal control and process control hindered innovation behaviors and outcomes (Park & Choi, 2014).

Besides governance choice, a growing body of literature has documented that appropriate parent–subsidiary and subsidiary–subsidiary social relationships enable MNEs to leverage knowledge effectively and achieve higher innovation outcomes. For example, communication and reciprocity among well-embedded internal actors of MNEs could facilitate knowledge flow and increase MNEs’ new patents (Asakawa et al., 2018; Gölgeci et al., 2019).

In addition, scholars are interested in the attributes of the structure of internal MNE networks, such as the different positions that internal units occupy within them. For example, Tsai (2001) researched two large MNEs and found organizational units can produce more new products than other internal units when they are central to the knowledge transfer network of the MNE. Tortoriello (2015) certified that in the internal MNE network, when individuals (e.g., employees) were located in rich structural holes, they could leverage knowledge from others more effectively toward the generation of new patents.

#### MNEs’ external network

MNEs tend to develop external networks with actors located outside their boundaries. Innovation requires knowledge diversity, and firms that are good at searching for and integrating different types of knowledge from external networks achieve better innovation outcomes than those that are not. Collaboration with partners (e.g., suppliers, customers, competitors, universities, or other institutions and governments) in external networks is one efficient way to provide MNEs with different types of knowledge to innovate. First, in partnership with global suppliers on activities such as offshoring of intermediate production (Valle et al., 2015) and R&D activities (Nieto & Rodríguez, 2011), MNEs can trigger new product innovations by acquiring knowledge about new components and materials and other low-cost and high-quality innovation resources. Nevertheless, researchers have found an inverted U-shaped relationship between R&D offshore outsourcing and product innovation, considering higher coordination and control costs, and overdependence on external knowledge from suppliers can cause neglect of the development of MNEs’ own knowledge stocks (Mihalache et al., 2012). Second, global customers provide MNEs with more diversified market knowledge, and MNEs’ customer-oriented strategy to consciously engage with foreign customers helps them keep track of their changing demands and generate innovative ideas (Harirchi & Chaminade, 2014; Hsieh et al., 2018). Third, engaging in collaboration with host country competitors could complement subsidiaries’ knowledge about local industry dynamics, local customer demands and preferences, and ways of handling local regulatory and social pressures, which would further help them create new products (Henttonen et al., 2016). Last, universities are also common partners for MNEs to cooperate with because they can synthesize knowledge and ideas from wide ranges of industries and governments, facilitating MNEs’ knowledge transfer, exploitation, and creation (Etzkowitz et al., 2005).

Another relevant discussion is about how the relationships between MNEs and external partners influence their innovation outcomes. Previous studies have revealed that subsidiaries’ well-embedded social relationships with local partners in the host country have helped them achieve more innovation. For example, Isaac et al. (2019) found that subsidiaries’ external relational embeddedness based on trust and adaptation was positively associated with subsidiaries’ new products and processes because supportive social relationships can help them overcome the liability of foreignness, adapt to local institutional environments discourage opportunism, reduce the sense of competitiveness and hostility, and encourage knowledge exchange and mutual learning. On the other hand, researchers have focused on the impact of governance of the global supply chain on MNEs’ innovation. They compared governance patterns in offshoring having to do with whether they were affiliates, in which affiliates could offer firms fewer appropriability problems and risks associated with knowledge transfer, which could have greater impacts on new products than interdependence (Steinberg et al., 2017). There is also the issue of how rigid-explicit behavioral controls and relational norms-based controls in the global supply chain influence MNEs’ innovation outcomes (Nieto & Rodríguez, 2011).

Furthermore, scholars are trying to provide a holistic understanding of the external MNE network composed of different types of actors, referring to its structural attributes. For instance, combined with institutional theory, Vasudeva et al. (2013) found that when the broker MNE locates in a corporatist country where collaboration and communication dominate (e.g., Japan), it is more capable of gaining innovation benefits from spanning structural holes in its global alliance networks. This is because such institutional environments can facilitate an MNE to manage a partnership better and to transfer and integrate knowledge more effectively than in the lower corporatist countries.

#### MNEs’ dual networks

MNEs’ subsidiaries are in essence embedded in dual networks and interact with multiple agents within internal and external networks. Recently, researchers have paid attention to how subsidiaries manage to innovate in the dual networks, most frequently using embeddedness theory (Achcaoucaou et al., 2014; Berry, 2018). For example, Berry (2018) compared how the embeddedness across a parent, host country, and third country knowledge networks influence foreign operation’s innovation outcomes. She found that high embeddedness within the parent firm motivated subsidiaries to extend the parent technology paradigm, thus leading to incremental innovation outcomes; while more distant knowledge from external networks can help subsidiaries generate more radical combinations of knowledge.

Meanwhile, one crucial question arises of how the interactions for subsidiaries’ dual embeddedness affects their own innovation outcomes, and studies reach no consensus. Some scholars have argued that dual embeddedness may create conflicts that inhibit subsidiaries’ innovation. For example, Andersson et al. (2005) found that parent firms’ direct controls of subsidiaries through expatriates could impede their external embeddedness in the host country, given that managers from the parent firm were unfamiliar with local business environments and need to take time to build local relationships, which prevented subsidiaries from creating new knowledge through external network embeddedness. Subsidiaries’ high embeddedness in the parent knowledge network could weaken the positive relationship between embeddedness in the host country and their radical innovation outcomes (Berry, 2018). Also, higher external embeddedness could lead to subsidiaries’ isolation from MNEs’ internal networks, thus hampering internal knowledge sharing and creation (Monteiro et al., 2008). Others have addressed the fact that the degrees of embeddedness in both networks could enhance each other, facilitating MNEs’ innovation outcomes. Subsidiaries’ embeddedness in external networks could strengthen their internal embeddedness by accelerating the transfer of newly acquired knowledge to be further exploited within MNEs (Ciabuschi et al., 2014); and subsidiaries that develop knowledge-intensive linkages with specific internal and external counterparts simultaneously could achieve higher innovative levels because they could explore complementarities and combine knowledge as sources of strategic assets (Figueiredo, 2011).

### Contingency factors

The relationship between internationalization and innovation is not linear but depends on a number of contingencies externally and internally to firms.

#### External country/region institutions

Innovation activity is highly embedded in national/regional institutional contexts, and the institutional environments have important implications for the costs of knowledge transaction and communication and for the difficulty of knowledge flow and absorption (Michael, 1990), which can further improve or hamper firms’ innovations during their internationalization process. As for the home country, Xie and Li (2018) adopted the institutional perspective to explore the moderating role of home regional institutional developments in the relationship between firms’ exports and new products. They found that well-developed innovation support institutions and market intermediaries at home helped firms gain more new products by facilitating knowledge flows and firms’ combination of overseas and local knowledge, but that market openness in home regions reduced firms’ access to innovation through exports because an open market could also provide them with foreign knowledge at home that could substitute for going abroad. Concerning the host country, it is generally believed that well-developed institutions in a host country can help firms achieve more innovation (Piperopoulos et al., 2018; Wu et al., 2018). Moreover, institutional distance represents an impediment to the transfer of technologies and combination of knowledge; large institutional distance can hinder firms’ new product outcomes through internationalization (Xie & Li, 2018).

#### Firm internal characteristics

First, innovation also depends on firms’ abilities to learn and integrate diverse knowledge and resources from multiple countries. On the one hand, scholars have discussed the impacts of firms’ dynamic capabilities on innovation outcomes, in which the sourcing capability can help firms effectively recognize and absorb knowledge in the host country, and combination capability helps combine internal and external knowledge and knowledge reconfiguration (Michailova & Zhan, 2015; Phene & Almeida, 2008). On the other hand, firms with strong absorptive capacity can better recognize and acquire diverse knowledge in international markets and internalize it into their own knowledge pool (Xie & Li, 2015; Yao et al., 2013). Moreover, Li et al. (2016) found that internationalization has a positive effect on new patents when small/medium-sized enterprises’ (SMEs) R&D or marketing capability is strong.

Second, firms’ resources including human capital, experience, and firm size, also can influence their innovation in the internationalization process. Discussions related to human capital mainly include managers and R&D personnel. For example, Elenkov and Manev (2009) found that senior expatriates’ visionary transformational leadership could influence subsidiaries’ innovation adoption rate because this kind of leadership promotes an organizational culture that encourages experimentation, risk taking, and freedom from punishment; Li et al. (2013) found that R&D expatriates from headquarters significantly contributed to innovation within subsidiaries located in an emerging country because R&D personnel deployed by the parent firm could facilitate internal transfer of high-quality technological knowledge within MNEs. Zhao (2006) further explored that MNEs could use internal knowledge linkages to protect their innovations in inadequate external institutions, since those innovations with closely knit internal technology structures are more difficult to imitate. Moreover, experience could facilitate MNEs’ local knowledge acquisition and lead to greater organizational learning in foreign markets, thus increasing their innovation outcomes, and researchers discussed previous international experience (Fu et al., 2018), prior R&D project experience (Demirbag & Glaister, 2010), and CEO industry experience (Nuruzzaman et al., 2019). In addition, a few authors have addressed the role of firm size in innovation, such as Golovko and Valentini (2014), who found that SMEs focused on product innovation when they entered export markets owing to their limited resources, narrower business scope, and difficulty dealing with foreign price discrimination, whereas large exporting firms were incentivized to pursue process innovation to improve their efficiency.

## Future research

In this section, we have further highlighted five important future research areas.

### Unpacking the dynamic and complex nature of internationalization entry in innovation

As discussed previously, the relationship between firms’ location diversity and innovation outcomes is yet to be settled, and literature regarding the relationship between temporal aspects of internationalization and innovation is still underdeveloped. We believe future researchers can dive deeply into unpacking the dynamic and complex nature of internationalization entry to fill the gaps, resolve prior inconsistencies, and move the discussion forward.

First, there is a need to examine how firms’ temporal aspect of foreign expansion (e.g., entry speed and rhythm) influences their innovation. On the one hand, how rapid entry into multiple countries influence the effectiveness of firms’ innovation deserves more attention. Despite the fact that high-speed foreign expansion can help firms quickly identify new opportunities and enjoy learning curve effects, it can also lead to time-compression problems that can significantly reduce the efficiency of learning from global markets (Vermeulen & Barkema, 2002; Yang et al., 2017). A close look at internationalization speed’s impact on firm innovation would be meaningful for firms to balance the tension associated with speed. On the other hand, entry rhythm refers to whether firms expand into multiple countries at an even pace, which is also worth further investigation. Shi and Prescott (2012) found that a relatively steady and regular entry pace could allow firms to allocate absorptive capacities for learning and accumulating knowledge, which could enhance their financial performance. Given such relevance, it is necessary to conduct empirical studies to certify the impact of entry rhythm on firms’ innovation outcomes. An unpacking of such dynamic natures of internationalization entry would not only explicitly highlights the importance of time in the relationship between internationalization and innovation, but also provide new angles to resolve the aforementioned inconsistent relationships between location diversity and innovation outcomes.

In addition to investigating temporal aspects of internationalization independently, it could be interesting to consider the combination of time and space to better explain the dynamics of internationalization entry. In firms’ location diversity and innovation research, the combination of time (i.e., when to enter) and location (i.e., where to enter) can be captured as entry order. For example, two Chinese multinational firms, Haier and Huawei, implemented location diversity strategies. Haier first entered the United States and then moved into Southeast Asia, while Huawei first entered Russia and then moved to German.^3^Considering that firms gradually build their capabilities through a series of events occurring over time (Beugelsdijk et al., 2017), it could be argued that even with both companies entering multiple countries, the learning differences associated with the entry order could result in heterogeneous innovation outcomes. Therefore, we believe it would be fruitful for IB scholars to further understand this dynamic process of firms’ internationalization and its impact on innovation.

Furthermore, the internationalization entry stage is not only dynamic but also complex, in which a single factor is not sufficient to explain the presence of a particular outcome (Beugelsdijk et al., 2017). Expanding into several countries is a complex strategy with temporal and spatial dimensions, intertwined with internationalization motivation, the economic state of the host country (emerging or developed), the difference between the host and home countries, and the associated entry mode decision. Different configurations of these factors may impose heterogeneous requirements on managers’ attention, firms’ capabilities, or routine developments and therefore may have distinctive influences on firms’ innovation. For this reason, a configurational approach to investigating a series of factors, including firms’ location diversity and their influence on innovation, would generate more insightful conclusions for future research. This would also respond to the recent call to take a configurational approach in explaining the complex IB phenomenon by using qualitative comparative analysis (Fainshmidt et al., 2020).

### Deepening the understanding of MNEs’ networks and innovation

In the preceding sections, we have outlined key strands that MNEs’ foreign operations within networks at the post-internationalization stage contribute to innovation outcomes. However, several gaps still remain.

First, it is necessary to discuss both the benefits and costs of embeddedness in MNEs’ operation networks when considering innovation. Despite a well-embedded relationship providing benefits for MNEs’ learning and innovation, embeddedness can also incur costs for MNEs’ innovation. For example, deeply embedding with one partner can lead to lock-in problems such as learning inertia and inadaptability (Jiang et al., 2018), which may further reduce MNEs’ innovation motivations, restrict access to new partners and knowledge, and inhibit the identification of new opportunities. However, the potential negative impacts on innovation are still underexplored. Future researchers should explore innovation barriers and threats arising from embeddedness in MNEs’ internal and external networks. More importantly, it would be insightful to investigate the boundaries of how embeddedness can effectively help MNEs transfer knowledge from internal units and external partners and enhance their innovation capability.

Second, there is also a need to look closely at the role of MNEs’ external network structures in their innovation. One critical direction would be capturing and examining the MNEs’ position in external networks, such as how MNEs’ proximity to the broker affects their innovation outcomes. Moreover, it is vital for future researchers to examine the role of the dynamic nature of MNEs’ network structure in innovation. MNEs’ network structure is not static, and MNEs can mobilize from the edge to centrality in an external network over time or vice versa, and can constantly connect and disconnect with partners in their networks as well (Cuypers et al., 2020). The dynamic nature of network structures can bring fluctuations in MNEs’ learning and knowledge acquisition, thus the question of how the dynamic nature of network structures influences MNEs’ innovation outcomes should be further unveiled.

Third, particularly for foreign subsidiaries, they are concurrently embedded in internal and external MNE networks. No doubt that they can leverage the complementary knowledge and resources from dual embeddedness (Figueiredo, 2011), but there are conflicts between both networks that are hard for subsidiaries to manage it well (Andersson et al., 2005). So, it is necessary to clarify how the interrelationships between subsidiaries’ ties to internal MNE networks and ties to non-MNE-owned actors affect their innovation. Moreover, scholars should also borrow wisdom from network literature to appropriately measure dual embeddedness and develop an in-depth understanding of dual networks, which can enrich our understanding of the nature of internationalization and innovation.

### Extending institutional impact on MNEs’ innovation

With regard to external institutional environments, previous scholars have discussed how institutions in both host and home countries, and the institutional distance, influence MNEs’ innovation in their internationalization processes. However, institutional environment is more complex and multi-layered, which deserves further research.

First, MNEs do not simply adapt to institutions; they can actively influence the institutions in which they are embedded (Cantwell et al., 2010). Thus it is important to elaborate on how the co-evolution of MNEs and local institutional environments affects their innovation behavior and outcome. For instance, when entering emerging economies with dynamic and underdeveloped institutional environments, DMNEs could engage in political activities such as lobbying or building political connections with local government leaders to obtain political legitimacy, access useful resources, and influence public policy (Jean et al., 2018). Therefore, viewing interactions between MNEs and institutional environments from a co-evolutionary perspective, future researchers could offer an informed understanding of how MNEs proactively engage in the development of host country institutions to gain more protections and profits for their innovation.

Second, given the increasing trend of de-globalization (e.g., global geopolitics and trade tensions), scholars should dive deeply into how MNEs innovate in such contexts. The trend of the anti-globalization movement could leave the global political environment more volatile. For instance, populist reactions could make host countries crowd out foreign firms via national protectionism, which further hampers MNEs’ overseas knowledge search (Lorenzen et al., 2020). A technology blockade could isolate foreign firms from local innovation resources (e.g., talents, technologies, and research institutions) (Luo, 2021). With this backdrop, MNEs’ global R&D alliance network could be disrupted, and the worst situation would be that firms barely form their global R&D collaboration. Therefore, scholars should pay more attention to ways de-globalization changes the patterns of firms’ innovation behaviors, especially for their international R&D sites and collaborations, and to how such changes influence their innovation outcomes.

Last, we need to explore the multi-dimensions and multi-layers of institutions to unfold their complex influences on MNEs’ innovation. On the one hand, the interactions of numerous dimensions of institutions (e.g., formal and informal institutional environments or regulative, cognitive, normative institutions) may facilitate or constrain MNEs’ knowledge-seeking, absorption, and application, thereby significantly hampering their innovation activities and outcomes. Therefore, future researchers should capture such multidimensional attributes of institutions to explore their impact on MNEs’ innovation. On the other hand, previous scholars have underestimated other levels of institutional environment (e.g., municipal) that could lead to large heterogeneity on a national or regional level in an institutional environment. It is necessary to capture multi-layer institutional environments including country, region and municipality levels, which can help us better understand the levels of institutional impact on innovation.

### Revising internationalization and innovation in the digitalization age

With the development of digitalization, changes have taken place in the global markets in terms of who participates, how business is done across borders, and where the economic benefits flow (Manyika et al., 2016). Such changes may provide a novel context to challenge and revise the relationship between internationalization and innovation in previous studies.

First, differently from traditional exports, digitalization could facilitate firms’ entry into global markets via digital platforms and make it possible to expand into multiple countries in a short time, such as selling manufactured goods via AliExpress or Amazon. This can decrease transaction costs, leaving more resources for firm innovation, while learning hurdles arise from distant interactions as well (Deng et al., 2022). Thus, the way firm internationalization through digital platforms influences firms’ innovations deserves attention. Beyond traditional border spanning of physical commodities, the development of digitalization is enabling firms to cross geographic boundaries virtually, such as by selling applications via the Apple Store. A key challenge for such firms is the concern over digital privacy, which has been attracting increasing attention from host governments, associated with tough regulatory constraints (e.g., General Data Protection Regulation),^4^ thereby inhibiting digital firms’ operations, knowledge sharing, and innovation activities across different countries. Therefore, a better understanding of host countries’ regulations on digital-related concerns may improve digital firms’ reactions to global innovation.

Second, it would be intriguing for future researchers to unpack the influence of digitalization on MNEs’ innovations through their networks. Digital technologies can help MNEs manage international operations within internal and external networks more efficiently, such as helping to develop virtual collaborative networks composed of individuals from different countries and various firms. MNEs can form virtual teams to coordinate and manage international innovative activities without physical colocation, reducing costs and inspiring innovative ideas. However, virtual teams face challenges as well, in terms of management and coordination issues and mutual trust building (Zeschky et al., 2014), which can impede MNEs’ promotion of innovation projects. Therefore, there is a need to explore how MNEs develop and manage virtual networks to make best use of digital technologies, thus achieving more innovation outcomes.

Last, in the digital age, scholars should further pay attention to how firms’ digital-related capability influences them to take advantage of resources in their internationalization process, thereby influencing their innovation. For example, given that MNEs may be swimming in the vast sea of data, big data analytic capability (BDAC) can help MNEs optimize operations and identify loyal and profitable customers, including BDA management, infrastructure, and talent-related aspects and capabilities (Agarwal & Dhar, 2014). MNEs with this capability are more likely to identify and seize innovation opportunities in the global markets. Inquiries addressing the moderating role of digital-related capabilities can be useful to enhance our understanding of the relationship between internationalization and innovation during the development of digitalization.

### Comparing DMNE’s and EMNE’s innovation

Over the past two decades, some MNEs from emerging economies have risen to leading positions in various industries. Despite the fact that Anand et al. (2021) concluded that EMNEs’ innovation is largely shaped by the challenges of catching up with advanced economies, comparisons between DMNEs’ and EMNEs’ innovation through internationalization have drawn little attention.

First, researchers should test and revise traditional IB theories that emerged from the DMNE experience for understanding the innovation of EMNEs. We know that DMNEs’ and EMNEs’ strategies at each stage of internationalization may be different, leading to divergent consequences in terms of innovation. For example, at the internationalization entry stage, DMNEs mostly follow the Uppsala model and first enter more proximate and similar countries, while EMNEs tend to exploit differences rather than similarities across countries by expanding into physically or economically distant countries (Ramamurti, 2012). It is worth exploring the distinction between DMNEs’ and EMNEs’ internationalization processes and linking such differences to understand their innovational activities or outcomes.

Second, another way to look at DMNEs’ and EMNEs’ differences is to compare government influences on both types of MNE. Compared with DMNEs, EMNEs are embedded in less-developed institutional environments that often lack political constraints, which are more likely to experience excessive governmental political meddling, discouraging EMNEs from innovating (Lazzarini et al., 2021). Nevertheless, some scholars have found that EMNEs could cultivate advantages difficult to replicate, such as strong political ties (Li et al., 2014), which could help them leverage government resources with more efficiency than DMNEs in support of their own innovations. Therefore, comparing the distinct impacts of government involvement, such as the degree and different types of government involvement (e.g., state ownership, government affiliations, government policies, and government attention) could enrich our understanding of how governments can best encourage and support different types of firms to innovate.

Third, it is generally believed that DMNEs have ownership advantages, such as superior technological competence and absorptive capacity, whereas EMNEs also have different kinds of ownership advantages that deserve further focus. For example, EMNEs have grounded understanding of customer needs in emerging markets, the ability to adjust and manufacture products and services at ultralow costs, and the capability to develop good enough products for local customers (Adarkwah & Malonæs, 2020; Ramamurti, 2012). Therefore, it should be meaningful to compare the ways EMNE and DMNE leverage their distinctive ownership advantages to achieve innovations they learn about from foreign countries.

## Conclusion

There is no doubt that internationalization has become one of the central channels for firms to develop innovation capabilities. Our paper is intended to provide a fruitful account of the past research and future agendas on this domain. We gravitated around two key research questions: *What is the current state of research on the relationship between internationalization and innovation? What implications for future research do our findings suggest?*

For the first question, we have provided a comprehensive picture regarding whether and how firms’ internationalization strategies and operations at each stage influence their innovation behaviors and outcomes, with the moderating role of external country/region institutions and firms’ internal characteristics. In addition, although past research has provided considerable insight, our review has identified several important gaps for future research, which we think warrant greater focus in the future. Table 6 and 7 are short summaries responding to both research questions.

**Table 6:** A Summary of the different themes in our review

Stage	Innovation behavior	Innovation outcome
Pre-internationalization	Firms’ various internationalization motivations can drive their international R&D activities	No direct linkages (Motivation should firstly influence behavior)
Internationalization entry	1. The advantages of a particular host country where firms locate can stimulate their R&D activities 2. The difference between the host country where firms locate and their home country affect their R&D activities 3. Non-equity entry mode in internationalization incentivizes firms to make R&D investments, whereas the role of equity entry mode in R&D activities is still controversial 4. Sustaining entry into global markets can help firms learn effectively, thereby motivating R&D investments, whereas the role of temporal behaviors behind it remains unclear	1. The advantages of a particular host country where firms locate can support innovation outcomes 2. The difference between the host country where firms locate and their home country has both benefits and costs in affecting innovation outcomes 3. Location diversity has both advantages and disadvantages; its impacts on innovation outcomes are inconsistent 4. Both non-equity and equity entry mode in internationalization can increase firms’ innovation outcomes 5. INVs that internationalize early have learning advantages to absorb and create knowledge, whereas discussion of entry speed is still lacking
Post-internationalization	1. Within MNE’s internal networks, excessive control from the parent firm can hinder subsidiaries’ innovation activities 2. Overdependence on knowledge from MNE’s external network can decrease internal R&D investments	1. Within MNEs’ internal networks, appropriate hierarchical governance and social relationships can help knowledge transfer and increase innovation outcomes 2. Within MNEs’ external networks, the type of collaboration partners and the relationships have great impacts on MNEs’ innovation outcomes 3. Within subsidiaries’ dual networks, the impacts of dual embeddedness on their innovation outcomes are mixed and underdeveloped
Contingency: External country/region factor	Institutional environments and institutional distances have important implications for the costs of knowledge transaction and the difficulty for knowledge combination, which can further improve or hamper firms’ innovation during their internationalization process	
Contingency: Firm internal factor	Firm capabilities, human resources, experience, and size can influence them to acquire, absorb, and apply knowledge to realize innovation in their internationalization process

**Table 7:** A summary of directions for future research

Future themes	Directions for future research
Internationalization entry and innovation	1. Temporal behaviors (e.g., speed and rhythm) of international expansion in innovation 2. The impact of entry order on innovation by considering time and space aspects of internationalization 3. Configurational approach to viewing the relationship between location diversity and innovation
MNEs’ network and innovation	1. Benefits and costs of external network embeddedness when considering innovation 2. The static and dynamic structure of MNEs’ external networks and innovations 3. The impacts of subsidiaries’ dual embeddedness on innovation
Institutional impact on MNE’s innovation	1. The coevolution between MNEs and local institutional environments and innovations 2. De-globalization and innovation 3. The multilayers and multi-dimensions of institutions and innovations
The relationship in the digitalization age	1. Digital internationalization and innovation 2. Digitalization can help restructure MNEs’ international networks, thus affecting innovation 3. Digital-related capabilities and MNEs’ innovation
DMNEs’ and EMNEs’ innovations	1. Revise traditional IB theories that emerged from DMNE experience to understand EMNEs’ innovation 2. Compare DMNEs and EMNEs’ home government influences on innovation 3. Compare DMNEs and EMNEs’ distinctive ownership advantages to achieve innovation

Despite the fact that our review has depicted a more comprehensive picture embracing key themes of the literature that have analyzed the relationship between internationalization and innovation, we still recognize limitations in the process of literature identification owing to different starting points, selection criteria, and author biases. Regardless, our review of the literature from 1989 through 2020 shows that there has been considerable progress in the past three decades on a number of issues in this domain, and ample research agendas may also play important roles in the future development of this relationship. We hope that our review facilitates related IB research that resonates with Buckley et al. (2017) to produce greater “impact, relevance, and a connection to the real world” (p. 1053).

## Footnotes

1 We formed two sets of keywords: the first having descriptive terms relating to internationalization and the second relating to innovation. When we run a topic-specific search in Web of Science, we combine the two sets to ensure that each keyword from one set was pairwise searched together with a keyword from the other. The first set includes internation* OR global* OR foreign* OR oversea* OR multination* OR “cross-border” OR “crossborder” OR “cross border” OR “geographic diversi*” OR “country diversi*”, and the second includes innovat* OR “R&D” OR “research and development” OR “knowledge transfer” OR “knowledge sourcing” OR “knowledge search” OR “knowledge flow” OR “knowledge mobility” OR “knowledge spillover” OR “knowledge creation” OR “knowledge acquisition” OR “knowledge network” OR “knowledge absorption” OR “knowledge capacity” OR “technology spillover” OR “technology transfer” OR “technological upgrading” OR “technology sourcing” OR “technology flow” OR “technological acquisition” OR “technological alliance” OR “technological capacity” OR “technological learning” OR “joint learning” OR “learning network” OR “organizational learning.”

2 The Web of Science allows us to enter all the keywords simultaneously in one go, while the Google Scholar has a limitation of word count so that we need to do multiple searches. More importantly, the retrieval algorithm is different. The paper can be retrieved on the Google Scholar as long as the keywords appear throughout the paper, whereas the Web of Science demands that keywords should exist in the section “Title,” “Abstract,” and “Keywords.”

3 Sources are available on official websites of Huawei and Haier

4
The General Data Protection Regulation is a regulation in EU law on data protection and privacy in the uropean Union and the European Economic Area
